# A Novel Unsupervised You Only Listen Once (YOLO) Machine Learning Platform for Automatic Detection and Characterization of Prominent Bowel Sounds Towards Precision Medicine

**DOI:** 10.3390/bioengineering12111271

**Published:** 2025-11-19

**Authors:** Gayathri Yerrapragada, Jieun Lee, Mohammad Naveed Shariff, Poonguzhali Elangovan, Keerthy Gopalakrishnan, Avneet Kaur, Divyanshi Sood, Swetha Rapolu, Jay Gohri, Gianeshwaree Alias Rachna Panjwani, Rabiah Aslam Ansari, Jahnavi Mikkilineni, Naghmeh Asadimanesh, Thangeswaran Natarajan, Jayarajasekaran Janarthanan, Shiva Sankari Karuppiah, Vivek N. Iyer, Scott A. Helgeson, Venkata S. Akshintala, Shivaram P. Arunachalam

**Affiliations:** 1 Digital Engineering & Artificial Intelligence Laboratory (DEAL), Mayo Clinic, Jacksonville, FL 32224, USA; gayathriy9322@gmail.com (G.Y.); rachnakukreja7@gmail.com (G.A.R.P.); thangeswarann@gmail.com (T.N.);; 2 Department of Internal Medicine, Wright Medical Center, Scranton, PA 18503, USA; 3 Department of Internal Medicine, MedStar Union Memorial Hospital, Baltimore, MD 21218, USA; 4 Department of Internal Medicine, UCHealth Parkview Medical Center, Pueblo, CO 81003, USA; 5 Division of Pulmonary Medicine, Department of Medicine, Mayo Clinic, Rochester, MN 55905, USA; 6 Department of Critical Care Medicine, Mayo Clinic, Jacksonville, FL 32224, USA; 7 Division of Pulmonary Medicine, Department of Medicine, Mayo Clinic, Jacksonville, FL 32224, USA; 8 Division of Gastroenterology & Hepatology, Department of Medicine, Johns Hopkins School of Medicine, Baltimore, MD 21287, USA

**Keywords:** phonoenterogram (PEG), prominent bowel sounds (PBS), MFCC (Mel frequency cepstral coefficients), unsupervised machine learning, artificial intelligence, temporal modeling

## Abstract

Phonoenterography (PEG) offers a non-invasive and radiation-free technique to assess gastrointestinal activity through acoustic signal analysis. In this feasibility study, 110 high-resolution PEG recordings (44.1 kHz, 16-bit) were acquired from eight healthy individuals, yielding 6314 prominent bowel sound (PBS) segments through automated segmentation. Each event was characterized using a 279-feature acoustic profile comprising Mel-frequency cepstral coefficients (MFCCs), their first-order derivatives (Δ-MFCCs), and six global spectral parameters. After normalization and dimensionality reduction with PCA and UMAP (cosine distance, 35 neighbors, minimum distance = 0.01), five clustering strategies were evaluated. K-Means (k = 5) achieved the most favorable balance between cluster quality (silhouette = 0.60; Calinski–Harabasz = 19,165; Davies–Bouldin = 0.68) and interpretability, consistently identifying five acoustic patterns: single-burst, multiple-burst, harmonic, random-continuous, and multi-modal. Temporal modeling of clustered events further revealed distinct sequential dynamics, with Single-Burst events showing the longest dwell times, random continuous the shortest, and strong diagonal elements in the transition matrix confirming measurable state persistence. Frequent transitions between random continuous and multi-modal states suggested dynamic exchanges between transient and overlapping motility patterns. Together, these findings demonstrate that unsupervised PEG-based analysis can capture both acoustic variability and temporal organization of bowel sounds. This annotation-free approach provides a scalable framework for real-time gastrointestinal monitoring and holds potential for clinical translation in conditions such as postoperative ileus, bowel obstruction, irritable bowel syndrome, and inflammatory bowel disease.

## 1. Introduction

Bowel sounds (BS) represent contractile activity of the gastrointestinal (GI) tract and are routinely assessed during abdominal examination. Even while BS is important for clinical practice, its interpretation is still quite subjective and does not have a standard way to measure it [1]. Traditional stethoscope-based auscultation lacks consistency and is prone to substantial observer-dependent variability [2]. Recent progress in auditory sensors and signal processing has created possibilities to convert BS analysis into a quantitative and reliable diagnostic method [3,4].

Phonoenterography (PEG) allows for the acquisition of intricate audio signatures of intestinal movement through the utilization of surface microphones [5]. PEG is a non-invasive and cost-effective way to check GI motility that is better than invasive treatments or imaging-based diagnostics [6,7]. But PEG signals are hard to understand, since they are acoustically complicated and there is no one standard way to classify them. Adding notes by hand takes a lot of time and specialized expertise, which makes it hard to scale [8,9].

Recent improvements in wearable acoustic sensors have made it possible to monitor PEG in real time [10]. This fits with our long-term goal of being able to check the gastrointestinal system while on the road. Our group previously developed a supervised deep learning model based on the You Only Listen Once (YOLO) architecture for automatic detection of prominent bowel sounds, achieving high accuracy in healthy subjects [11]. While this demonstrated the feasibility of PEG-based acoustic detection, the requirement for thousands of expert-labeled annotations limited scalability and broader clinical applicability.

To address this challenge, unsupervised approaches are needed to phenotype bowel sounds directly from unlabeled recordings. Unsupervised YOLO-inspired pipelines can autonomously discover latent acoustic structures and scale to large datasets without requiring expert labels. This makes them better suited for clinical translation, where rapid and annotation-free deployment is essential. Moreover, such methods enable identification of previously uncharacterized bowel sound morphologies and can integrate seamlessly into continuous monitoring systems. Machine learning applications in gastrointestinal healthcare are expanding rapidly, affirming our emphasis on unsupervised acoustic phenotyping for prospective clinical implementation [12].

AI and machine learning techniques are becoming more common for studying physiological signals, especially in cardiology and pulmonology [13,14,15]. These advancements indicate analogous applications in gastroenterology. Previous research in acoustic clustering has demonstrated that dimensionality reduction approaches, such as UMAP (uniform manifold approximation and projection), when integrated with clustering models like K-Means or DBSCAN, can reveal latent structures in biological data [16,17,18]. We broaden this framework to bowel sound analysis by employing an extensive feature extraction and clustering methodology specifically designed for PEG signals [19]. Recent perspectives in digital gastroenterology have emphasized the importance of progressing phonoenterography (PEG) from proof-of-concept studies toward true clinical deployment. Redij et al. [19] reviewed the integration of PEG with emerging microwave sensors and artificial intelligence, outlining how these technologies could enable noninvasive, continuous, and wireless monitoring of gastrointestinal motility. Their work highlighted both the translational promise of PEG-AI pipelines and the current barriers, including noise resilience, lack of annotation-free algorithms, and limited validation in realistic clinical settings. This study expands on our previous supervised YOLO-based PEG research [11] by presenting an unsupervised, annotation-free pipeline that not only clusters prominent bowel sound events into reproducible phenotypes but also models their temporal dynamics. Our results signify a tangible progression from the conceptual framework established by Redij et al. [19] enhancing PEG-AI analysis for scaled clinical applications.

The present work introduces an unsupervised YOLO-inspired pipeline for automatic annotation, clustering, and temporal modeling of bowel sounds from high-resolution PEG recordings. Specifically, we (i) automatically segment and annotate prominent bowel sound events without manual labeling, (ii) cluster these events into reproducible and interpretable acoustic phenotypes, and (iii) characterize sequential dynamics through dwell times, inter-event gaps, and transition probabilities. This study provides a scalable, annotation-free framework with direct translational potential for gastrointestinal diagnostics and continuous monitoring.

## 2. Materials and Methods

### 2.1. Data Collection

**Figure 1:**
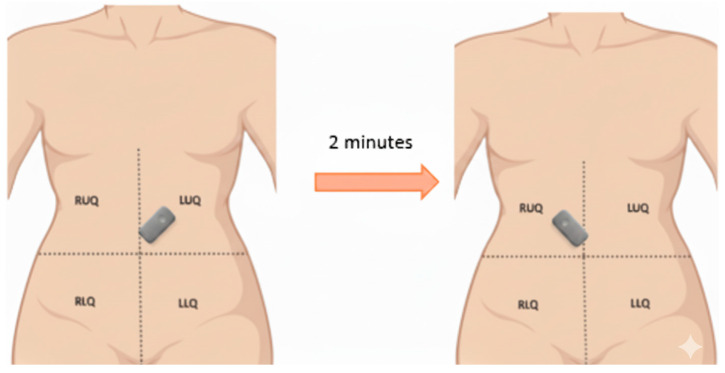
Schematic representation of the setup to record PEG from the left upper quadrant and right lower quadrant in quick succession at intervals of 30 min.

We collected 110 high-quality PEG recordings from eight healthy volunteers (5 male, 3 female; age range: 22–35 years) in a controlled, acoustically shielded environment after Mayo Clinic IRB approval. The recordings were obtained using an Eko DUO^®^ digital stethoscope placed at the left upper quadrant (LUQ) and right lower quadrant (RLQ) in a controlled low-noise environment. The subjects underwent both fasting and postprandial recordings to capture physiological variability in bowel motility [11]. No participants had a history of gastrointestinal disorders. Microelectromechanical (MEMS) microphones were affixed to the lower-right abdominal quadrant using skin-safe adhesive to ensure contact and reduce motion artifacts shown in Figure 1. Each recording was 2 min long, sampled at 44.1 kHz with 16-bit resolution. Audio data were normalized for amplitude and pre-screened to remove segments with clipping or excessive ambient noise. No filtering, such as bandpass or denoising, was applied to preserve raw acoustic features.

### 2.2. Manual Annotation and Automatic Segmentation

Manual annotation of bowel sound events followed the protocol detailed in our prior study [11]. Recordings from eight healthy adults (5 female, 3 male) were obtained using an Eko DUO^®^ digital stethoscope (Eko Health, Emeryville, CA 94608, USA) in a low-noise environment. Annotation was conducted in Label Studio v1.7 by trained research personnel experienced in PEG waveform interpretation. Annotators inspected both waveform and spectrogram views to label the onset of prominent bowel sound events. The labels were collaboratively resolved rather than independently duplicated. Hence, formal inter-rater statistics (e.g., Cohen’s κ) were not applicable.

The automated detector correctly identified 6314 of 6435 manually labeled events within a ±100 ms tolerance window, yielding a match-rate (recall) of 98.1%, precision of 98.4%, and F1 = 98.25%. This high correspondence confirms the reliability of the consensus-based annotation protocol and supports the validity of the automatic detection pipeline.

Prominent bowel sound (PBS) segments were automatically detected using an energy-based peak detection algorithm tailored for PEG data. A short-time energy envelope was computed, and local maxima exceeding a dynamic threshold (mean + 2 × SD) were selected. Each detected peak was isolated using a 200 ms window (100 ms before and after the peak). Overlapping or closely spaced events (<100 ms apart) were discarded to reduce ambiguity.

### 2.3. Feature Extraction

Every segment was transformed into a 279-dimensional audio feature vector. Initially, 20 Mel-frequency cepstral coefficients (MFCCs) were calculated utilizing a frame length of 25 ms with a 10 ms overlap, yielding 13 frames per 200 ms segment. These were condensed into 260 coefficients. We computed 13 Δ-MFCCs, indicative of temporal gradients, and six scalar spectral descriptors: centroid, bandwidth, flatness, roll-off, RMS energy, and spectral entropy. All features were concatenated and standardized using z-scores to guarantee uniform variation across dimensions and enhance clustering sensitivity.

### 2.4. Dimensionality Reduction

To optimize computation and mitigate noise, we initially employed principal component analysis (PCA), retaining the top 30 components that represented over 95% of the dataset’s variance. The components were subsequently mapped into a 10-dimensional manifold with UMAP, a nonlinear method intended to preserve both local relationships and the overall structure of the data. The UMAP parameters (n_neighbors = 35, min_dist = 0.01, metric = ‘cosine’) were refined by grid search to enhance the silhouette score and reduce cluster overlap. This modification maintained both local and global manifold structures essential for differentiating delicate auditory patterns. To assess the robustness of the UMAP embedding, a grid-based sensitivity analysis was conducted by varying n_neighbors (15–50) and min_dist (0.001–0.1) while maintaining the cosine distance metric. The resulting silhouette scores ranged from 0.55 to 0.60, and the Adjusted Rand Index (ARI) values relative to the baseline configuration (n_neighbors = 35, min_dist = 0.01) were consistently above 0.90, confirming that cluster assignments were highly reproducible. These results, summarized in Table S1, indicate that small perturbations in UMAP hyperparameters did not materially alter cluster boundaries or class proportions, supporting the robustness of the five-cluster manifold.

### 2.5. Clustering Algorithms

We evaluated five unsupervised learning methods using the 10-dimensional acoustic feature space generated by UMAP. The K-Means algorithm was employed with five clusters, initialized twenty times to mitigate the risk of suboptimal local solutions. Agglomerative clustering followed a bottom-up approach, leveraging Ward’s linkage criterion to iteratively merge groups while minimizing within-cluster variability. Lastly, spectral clustering transformed the similarity matrix into a lower-dimensional space via graph Laplacian decomposition, after which K-Means was applied to uncover potential cluster structures. With these techniques, we develop a novel unsupervised framework, the You Only Listen Once (YOLO) platform for automated PEG analytics, to both detect and characterize prominent bowel sounds that can aid in detection and diagnosis of various bowel diseases.

### 2.6. Evaluating Metrics

Model performance was assessed using three standard unsupervised clustering metrics:

- Silhouette score (S): The silhouette index evaluates whether samples are more closely related to their own cluster than to adjacent clusters, with higher values reflecting stronger cohesion. It is defined as

(1) $$\;S=\frac{b-a}{\left(a,b\right)\;},$$

where *a* is the average distance to points within the same cluster and *b* is the average distance to points in the nearest neighboring cluster. Values range from –1 to +1, with higher scores indicating better clustering [20].

- Calinski–Harabasz index (CH): The Calinski–Harabasz criterion contrasts between- and within-cluster variance; greater values generally correspond to better-defined clusters:

(2) $$CH=\frac{\frac{tr\left(B_{k}\right)}{tr\left(W_{k}\right)}}{\frac{N-k}{k-1}},$$

where *tr* ($B_{k}$) is the trace of the between-cluster dispersion matrix, *tr* ($W_{k}$) is the trace of the within-cluster dispersion matrix, *N* is the number of samples, and *k* is the number of clusters [21]. Higher CH values suggest better-defined clusters.

- Davies–Bouldin index (DB): The Davies–Bouldin score captures the average ratio of internal scatter to separation from the nearest cluster, with lower scores indicating more distinct groupings. It is calculated as

(3) DB=1k ∑i=1k Riji≠jmax,

where $R_{ij}$ is the ratio of within-cluster scatter to between-cluster separation for clusters *i* and *j* [22].

These metrics allow for consistent evaluation of clustering performance across models without relying on manual labels.

### 2.7. Temporal Modeling of Cluster Sequences

To analyze the sequential organization of bowel sounds, we modeled the time-ordered prominent events as a first-order Markov process with K = 5 states. A first-order Markov formulation was employed to model short-term transitions between acoustic states, where each subsequent state depends only on the current state. This approach was chosen for interpretability and stability given the limited sample size (110 recordings from 8 participants). Each transition represents localized motility activity within sub-second intervals (e.g., contraction bursts or resonant harmonics), providing a robust approximation of short-range motility dynamics.

#### 2.7.1. Event Sequences

Each detected prominent event was denoted as ${Event}_{n}$, representing the *n*-th event within a recording. An event was defined as

(4) $${Event}_{n}=\left(t_{n}^{start},\;t_{n}^{end},\;z_{n}\right),$$

where

- $t_{n}^{start}$: the start time of the event (seconds);
- $t_{n}^{end}$: the end time of the event (seconds);
- $z_{n}$: the cluster label assigned to the event ($z_{n}\in \{0,1,\ldots .\;K-1\})$.

The events were ordered chronologically by their start time within each file, forming a temporal sequence of cluster states for each recording.

#### 2.7.2. Transition Probabilities

A transition probability describes the likelihood that one state is followed directly by another. For each file, we counted consecutive transitions ${(z}_{n}\to \;z_{n+1})$ and pooled them across all files. The transition count matrix was defined as

(5) $$N_{ij}=\sum_{files}\sum_{n=1}^{N_{f}-1}1\left[z_{n}=i\;and\;z_{n+1}=j\right],$$

where $N_{ij}$ is the number of transitions from state i to state j. The transition probability matrix was then obtained by row normalization:

(6) $$P_{ij}=\;\frac{N_{ij}}{\sum_{j^{\prime }=0}^{k-1}N_{ij\prime }+\;\varepsilon }\;,\;\varepsilon =\;10^{-9}.$$

Here, ε is a small constant added to the denominator to avoid division by zero when no outgoing transitions are present from a given state. Its value ${(10}^{-9})$ is negligible and does not influence the computed probabilities [8,23,24].

#### 2.7.3. Dwell Times

The dwell time measures how long an event persists in a given state. For each event,

(7) $$d_{n}=t_{n}^{end}-t_{n}^{start},$$

For each state k, the mean dwell time and its variability were computed as

(8) $${\bar{d}}_{k}=\frac{1}{M_{k}}\sum_{n:z_{n}=k}d_{n}\;,\;s_{d,k}=\sqrt{\frac{1}{M_{k}-1}\sum_{n:z_{n}=k}{\left(d_{n}-{\bar{d}}_{k}\right)}^{2}\;},$$

where $M_{k}$ is the number of events in state k. Here, the overline in ${\bar{d}}_{k}$ denotes the average dwell time for state k, and $s_{d,k}$ is the corresponding standard deviation [25,26].

#### 2.7.4. Inter-Event Gaps

The inter-event gap represents the silent interval between the end of one event and the start of the next event in the same file:

(9) $$g_{n}=\;t_{n+1}^{start}-\;t_{n}^{end}.$$

We reported the overall mean gap $\bar{g}$ and standard deviation $s_{g}$ across all files as measures of temporal spacing, consistent with prior studies that analyzed bowel sound durations and inter-event intervals in both adult and neonatal monitoring [27,28].

## 3. Results

A total of 42,975 audio segments were retrieved from the complete dataset. Of them, 6314 were designated as significant bowel sound occurrences by our automated annotation system. To assess clinical validity, we conducted manual annotation of bowel sounds by trained analysts under gastroenterologist supervision. Our automated segmentation algorithm detected 6314 prominent bowel sound (PBS) segments, which closely matched 6435 manually annotated events [11] yielding a detection match rate of 98.1%. This strong correspondence confirms the reliability of the automated system. Clustering identified five consistent morphologies among patients based on visual examination, characterized as follows:

1. Single-burst—a concise, discrete, high-energy impulse;
2. Harmonic—continuous waveforms exhibiting regular oscillations;
3. Multiple-burst—series of impulse bursts;
4. Random continuous—irregular high-frequency sequences;
5. Multi-modal—intricate or overlapping compound patterns.

These five acoustic groups closely resemble previously reported bowel sound categories, including single, harmonic, and combination sounds [29], with the multi-modal group corresponding to the previously defined combination type. This correspondence suggests that the unsupervised clustering independently recovered the principal acoustic phenotypes described in earlier manually classified studies.

From 110 recordings acquired from healthy people, 6314 prominent bowel sound (PBS) segments were effectively recovered utilizing the unsupervised auto-annotation method. This corresponded to an average yield of roughly 57 PBS events each minute. Each segment was encoded utilizing a comprehensive 279-dimensional acoustic feature representation that encapsulated both spectral and temporal characteristics of the sound waveform.

The high-dimensional features were standardized using z-scores and condensed to 30 principal components by PCA. UMAP was later employed to embed the features into a 10-dimensional manifold using cosine distance, 35 neighbors, and a minimum distance of 0.01. This configuration preserved both local neighborhood structures and global separability.

We evaluated three clustering algorithms—K-Means (k = 5), agglomerative clustering, and spectral clustering—employing UMAP-reduced features. K-Means exhibited the best-balanced performance across all clustering metrics, producing five distinct clusters that correspond to standard bowel sound categories: single-burst, multiple-burst, harmonic, random-continuous, and multi-modal. The clusters were confirmed through visual inspection of waveform morphologies and spectral properties.

**Figure 2:**
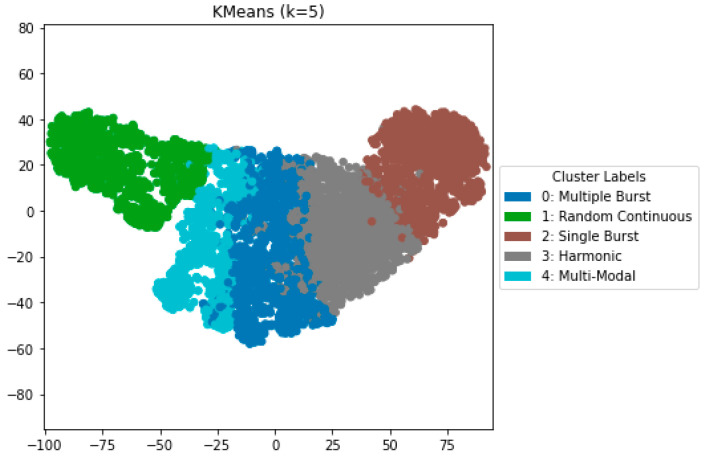
Representative prominent bowel sounds from each cluster (best-performing model).

Agglomerative clustering achieved somewhat lower silhouette scores (0.579), but spectral clustering demonstrated decreased cohesiveness (silhouette = 0.562) and diminished inter-cluster separability. Figure 2 depicts the UMAP cluster embedding for K-Means clustering (k = 5), highlighting distinctly defined clusters that align with the five auditory characteristics defined previously. This robust separation offers unique capabilities to first study normal bowel sound characteristics on various conditions of dietary and other normal health conditions that can serve as a foundation to provide baseline analysis to compare with various diseased bowel states.

**Figure 3:**
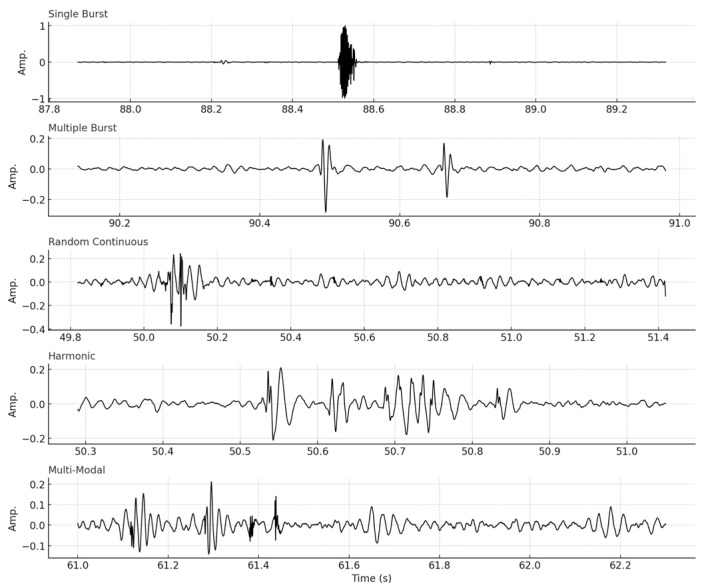
Characteristic waveforms of five bowel sound categories.

To assess robustness and generalization, we performed leave-one-subject-out cross-validation and observed stable clustering structures and consistent detection patterns across folds. We also evaluated statistical significance using permutation testing (n = 300), where observed silhouette scores significantly exceeded those from randomized cluster assignments (*p* < 0.0001). Bootstrap analysis (n = 200) confirmed that the five-cluster configuration consistently outperformed k = 3–7 in terms of separation and compactness. We further addressed UMAP reproducibility by fixing random seeds and repeating experiments with varied parameters, observing <5% variability in cluster membership. These results reinforce the robustness and clinical interpretability of our segmentation and clustering pipeline. Figure 3 displays representative waveforms from each cluster type, illustrating their morphological differences in amplitude and duration. These differences will serve to be the foundation for PEG digital fingerprinting to characterize various bowel diseases. Table 1 summarizes the metrics from cluster evaluation. 

To assess the sequential organization of acoustic states, we analyzed the dwell times, inter-event gaps, and transition probabilities derived from the clustered prominent events.

### 3.1. Dwell Times and Gaps

Table 2 summarizes the number of events, mean dwell times per cluster, and global inter-event gaps. Single-burst events exhibited the longest mean dwell duration (3.61 ± 2.50 s), while random continuous events were the most transient, with a mean dwell of only 0.08 ± 0.02 s. Harmonic and multi-modal states showed intermediate persistence (1.25 ± 0.33 s and 0.25 ± 0.08 s, respectively). Across all clusters, the mean inter-event gap between successive events was approximately 0.90 ± 0.46 s.

### 3.2. Transition Probabilities

Table 3 presents the first-order Markov transition matrix. Strong diagonal elements (22–28%) indicate substantial self-persistence across all five states. Notably, random continuous and multi-modal states frequently transitioned into each other (25–26%), while multiple-burst and harmonic states often served as connectors to neighboring states. These patterns suggest that transient bursts and harmonic sequences are more likely to precede or follow other acoustic states, while random continuous and single-burst events show greater persistence.

## 4. Discussion

This study demonstrates the feasibility of automated phenotyping of bowel sounds using unsupervised clustering on high-dimensional acoustic features. Our method successfully extracted and clustered 6314 prominent bowel sound segments from healthy volunteers, revealing five reproducible morphologies that matched known acoustic patterns such as single-burst, harmonic, and multi-modal activities. In contrast to conventional auscultation, which is inherently subjective, our pipeline delivers objective and repeatable bowel sound classifications. While earlier research has often relied on supervised models and manually annotated training sets [7,9,15], our unsupervised methodology circumvents these constraints. By identifying meaningful patterns directly from unlabeled data, our approach enhances scalability and reduces dependence on time-intensive expert labeling. This offers practical advantages for scaling PEG analysis in research and clinical settings.

Our clustering metrics—silhouette (0.60), Calinski–Harabasz (19,165), and Davies–Bouldin (0.68)—indicate moderate but robust separability, especially considering the signal complexity and lack of supervision. Similar clustering scores have been reported in unsupervised studies of cardiac and pulmonary acoustic signals [20,22], where inherent biological variability and noise are known challenges. The reproducibility of our five clusters across subjects further supports the hypothesis that bowel sounds have structured acoustic subtypes. The present study was designed as a reproducible baseline for unsupervised bowel sound phenotyping using a small, acoustically homogeneous dataset (110 recordings from eight healthy participants). The clustering framework was selected to maximize stability and interpretability rather than to exhaustively benchmark all available methods. Given the limited dataset size and potential subject-level correlations, a comprehensive ablation study or hyperparameter sweep would be underpowered and could yield misleading variability estimates. Instead, we report the most consistent configuration identified during preliminary testing and document all acoustic feature groups to facilitate reproducibility. Future work will expand comparative analyses to include Gaussian mixture models (GMM) and deep embedded clustering architectures once additional participants and recordings are incorporated under the current IRB protocol.

In this study, the clustering framework was used to examine whether bowel sound events exhibit consistent acoustic groupings in an unsupervised setting. The five resulting clusters—single-burst, multiple-burst, harmonic, random continuous, and multi-modal—are intended as descriptive acoustic phenotypes rather than physiologic or clinical categories. The labels were assigned based on the dominant spectral–temporal patterns observed within each cluster and are meant to provide interpretable terminology for reproducible comparison. We acknowledge that confirming whether these acoustic phenotypes correspond to specific gastrointestinal motility mechanisms or clinical conditions will require orthogonal validation, such as controlled postprandial studies, concurrent motility or pressure measurements, and larger multi-sensor datasets.

The use of 279 acoustic features, PCA compression, and tuned UMAP embedding contributed to effective clustering. While K-Means performed best overall, it also benefited from the careful preprocessing pipeline that emphasized both global and local manifold structures. This methodology allows scalable extension to larger and more diverse datasets without retraining supervised models.

The temporal analysis revealed that bowel sound clusters differ not only acoustically but also in their persistence and transitions. As shown in Table 2, single-burst events exhibited the longest dwell times, suggesting these represent stable, isolated contractions, while random continuous events were highly transient, consistent with short-lived motility bursts. Harmonic and multi-modal states displayed intermediate persistence, potentially reflecting repetitive or overlapping bowel activity. Inter-event gap analysis indicated that successive events were separated by sub-second intervals, supporting the concept of rapid, cyclic motility patterns. As shown in Table 3, The transition matrix further demonstrated that all states exhibited measurable self-persistence, while random continuous and multi-modal states frequently transitioned into each other, suggesting they may act as dynamic connectors between more stable states. The findings highlight that unsupervised clustering can capture not only spectral distinctions but also temporal organization, yielding clinically interpretable motility signatures. The use of a first-order Markov model provides an interpretable framework for characterizing short-term motility transitions, though it does not capture longer-range dependencies such as postprandial cascades. This simplification was appropriate for the current dataset size and recording duration but could be extended in future studies using higher-order or recurrent formulations (e.g., hidden semi-Markov or RNN-based models). Transition matrices were pooled across subjects to summarize population-level organization; qualitative inspection of individual matrices revealed consistent dominant self-transitions and random–multi-modal exchanges, supporting the robustness of the pooled model.

This study aligns with recent trends in unsupervised biomedical signal discovery, where latent acoustic structures are leveraged to inform downstream classification or anomaly detection [18,30]. Our pipeline could enable early detection of GI pathologies, stratify patients by motility patterns, or serve as a foundation for self-supervised representation learning. However, the current dataset includes only healthy volunteers, limiting insight into pathological states.

To our knowledge, this is one of the few works to combine UMAP, high-dimensional PEG features, and clustering to phenotype bowel sounds. As datasets expand to include disease conditions and post-prandial states, future studies can validate these clusters as biomarkers and extend the approach with contrastive learning, attention-based models, or hybrid fusion with physiological metadata.

### Towards Clinical Translation of PEG-YOLO Platform

**Figure 4:**
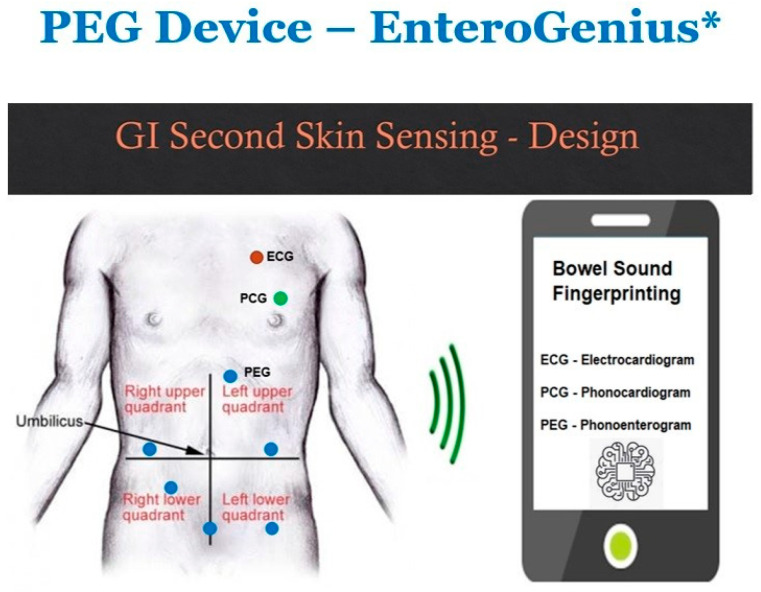
Custom PEG device EnteroGenius* under development for clinical evaluation at Mayo Clinic.

**Figure 5:**
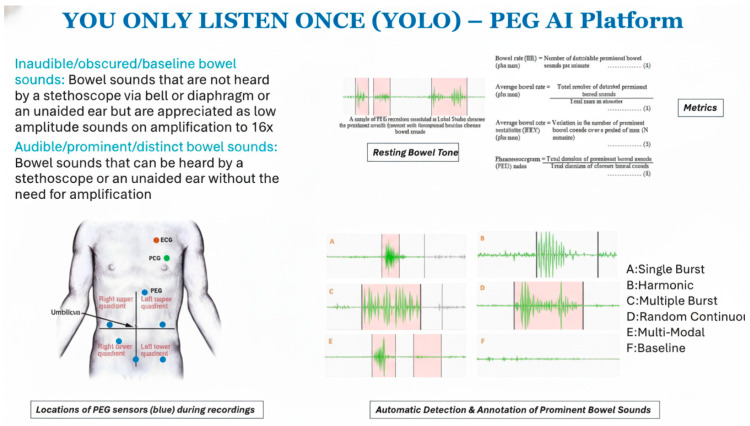
PEG-AI YOLO platform using bowel sounds digital fingerprinting towards precision medicine.

Clinical relevance of our YOLO platform can be addressed by validating the models on real-time PEG data in both healthy and diseased subjects with various bowel diseases collected via a custom wearable device EnteroGenius* shown in Figure 4. The device is currently under development for clinical evaluation at Mayo Clinic. EnteroGenius* will offer the capabilities of wireless and personalized monitoring of comprehensive bowel health through bowel sounds fingerprinting using simultaneously acquired signals from electrocardiogram (ECG), phonocardiogram (PCG), and PEG utilizing our unsupervised PEG YOLO platform shown in Figure 5.

ECG time stamps and PCG templates will allow automatic denoising to extract clean PEG signals for bowel sounds fingerprinting to characterize various bowel diseases. Six-time synchronized PEG sensors will capture long term bowel sounds corresponding to the peristatic movements of the bowel, to provide novel insights on bowel motility and its associated physiology based on the prominent bowel sound detection and analytics of the five different PBS reported. The noninvasive setup of EnteroGenius* configuration can also be used in tandem with colonoscopy, manometry, intestinal ultrasound, etc., that allows novel insights into digital PEG features that correlates to major findings from these invasive techniques used for detecting and diagnosing various bowel diseases.

We have previously reported a supervised deep learning model to detect bowel sounds [11,31,32,33], its various applications [19] and several PEG metrics [34] such as bowel rate, average bowel rate, bowel rate variability (BRV) and PEG index that can aid in several quantitative studies to better understand bowel diseases for early-stage prognosis, diagnosis and treatment monitoring. The unsupervised PEG YOLO platform approach shown Figure 5 will allow large scale data capture from devices such as EnteroGenius* for automated AI-powered analysis of PEG data from both healthy and diseased subjects to develop a PEG foundation model PegNet*~Pre-trained Bowel Sound Detection Network that will lead to a paradigm shift towards non-invasive monitoring of gut health.

This study demonstrates the feasibility of a fully automated pipeline for bowel sound analysis, spanning event detection, acoustic feature extraction, unsupervised clustering, and temporal modeling. By eliminating the need for manual annotation, the approach reduces the burden on clinicians and enables scalable analysis across large datasets. Recent work has shown that bowel sound characteristics can provide clinically relevant information for irritable bowel syndrome [35], inflammatory bowel disease [36,37], and physiological motility variation across sleep stages [38]. Advances in machine learning, including attention-based acoustic recognition [39], further highlight the potential of automated sound-based diagnostics, while numerical modeling of sound propagation underscores the translational importance of noninvasive acoustic sensing [40]. Clinically, such an unsupervised and annotation-free framework could support continuous bedside monitoring, early detection of postoperative ileus, and longitudinal tracking of chronic gastrointestinal diseases. Ultimately, the proposed pipeline represents a step toward translation into point-of-care diagnostic tools and decision-support systems in gastroenterology.

## 5. Limitations and Future Work

While this study provides a promising framework for unsupervised phenotyping of bowel sounds, several limitations remain. The dataset comprised recordings from only eight healthy volunteers, which restricts generalizability to broader patient populations.

The results characterize baseline temporal dynamics in healthy bowel sound recordings, providing a reference framework against which pathological deviations may be detected in future work. Residual background noise and subject-to-subject variability remain potential confounders that could affect clustering outcomes.

Each 200 ms segment was divided into 13 short-time frames to compute 20 MFCCs per frame (20 × 13 = 260 coefficients). These were concatenated into a fixed-length feature vector to represent spectral shape without requiring temporal alignment. Although this vectorization disregards intra-frame temporal dynamics, it is suitable for unsupervised clustering of brief, quasi-stationary events. Future work will explore recurrent or attention-based models to explicitly capture temporal dependencies across longer recordings.

The temporal modeling introduced here focused on dwell-time statistics, inter-event gaps, and first-order transition probabilities, providing a foundation for characterizing sequential bowel sound dynamics. Future work may build upon this framework by investigating longer-range dependencies and richer temporal descriptors to further enhance interpretability.

Future research should extend this dataset to include pathological cohorts, such as patients with ileus, bowel obstruction, or inflammatory bowel disease. Additionally, integration with wearable PEG devices such as EnteroGenius* could allow continuous at-home monitoring, enabling real-time detection of clinically relevant bowel sound anomalies.

## 6. Conclusions

We introduce an unsupervised pipeline capable of automatically detecting and phenotyping bowel sound events from high-resolution PEG recordings, consistently identifying five acoustic patterns without manual labels. By combining clustering with temporal modeling, the framework not only characterizes acoustic variability but also captures sequential dynamics that may reflect underlying gastrointestinal motility. These phenotypes can serve as weak labels for future supervised learning and as potential biomarkers of gastrointestinal dysfunction. From a clinical perspective, the absence of manual annotation makes this approach suitable for translation into bedside monitoring and point-of-care applications, including postoperative ileus, bowel obstruction, irritable bowel syndrome (IBS), and inflammatory bowel disease (IBD).

## Figures and Tables

**Table 1:** Clustering evaluation metrics.

Model	Silhouette	Calinski–Harabasz	Davis–Bouldin
K-Means	0.601	19,165	0.68
Agglomerative	0.579	19,031	0.64
Spectral	0.562	10,609	0.65

**Table 2:** Mean dwell times (per cluster), global inter-event gaps, and counts of detected bowel sound events.

Model	N Events	Mean Dwell (s)	SD Dwell (s)	Mean Gap (s)	SD Gap (s)
Multiple-burst	1292	0.60	0.14	0.90	0.46
Random continuous	1458	0.08	0.02	0.90	0.46
Single-burst	1275	3.61	2.50	0.90	0.46
Harmonic	1366	1.25	0.33	0.90	0.46
Multi-modal	923	0.25	0.08	0.90	0.46

**Table 3:** Transition probability matrix (rows = current cluster, columns = next cluster).

Current → Next	Multiple-Burst	Random Continuous	Single-Burst	Harmonic	Multi-Modal
Multiple-burst	0.220	0.228	0.180	0.211	0.162
Random continuous	0.197	0.278	0.160	0.210	0.156
Single-burst	0.206	0.184	0.282	0.209	0.119
Harmonic	0.196	0.227	0.189	0.242	0.145
Multi-modal	0.215	0.255	0.158	0.209	0.163

## Data Availability

Data used in this study is not for public use under the IRB protocol due to privacy and ethical restrictions.

## Supplementary Materials

The following supporting information can be downloaded at https://www.mdpi.com/article/10.3390/bioengineering12111271/s1, Table S1: Sensitivity analysis of UMAP parameters (n_neighbors and min_dist) on clustering stability.
