# Supplementary material for: A Novel Unsupervised You Only Listen Once (YOLO) Machine Learning Platform for Automatic Detection and Characterization of Prominent Bowel Sounds Towards Precision Medicine
Source: Bioengineering (Basel). 2025 Nov 19;12(11):1271. doi: 10.3390/bioengineering12111271 (PMC12650524; doi:10.3390/bioengineering12111271)
Supplement: Supplementary file 1 [file bioengineering-12-01271-s001.zip › bioengineering-3939132-supplementary.pdf]

Table S1. Sensitivity analysis of UMAP parameters (n_neighbors and min_dist) on clustering stability.

N_neighbors	min_dist	Silhouette	ARI vs Baseline
15	0.001	0.585	0.495
15	0.01	0.581	0.495
15	0.05	0.568	0.491
15	0.1	0.548	0.933
25	0.001	0.591	0.955
25	0.01	0.584	0.959
25	0.05	0.571	0.970
25	0.1	0.556	0.951
35	0.001	0.596	0.991
35	0.01	0.601	1.0
35	0.05	0.573	0.969
35	0.1	0.557	0.945
45	0.001	0.592	0.980
45	0.01	0.587	0.972
45	0.05	0.572	0.954
45	0.1	0.554	0.927
50	0.001	0.596	0.981
50	0.01	0.589	0.976
50	0.05	0.575	0.958
50	0.1	0.558	0.934
